# Analysis of a Commercial Insurance Policy to Deny Coverage for Emergency Department Visits With Nonemergent Diagnoses

**DOI:** 10.1001/jamanetworkopen.2018.3731

**Published:** 2018-10-19

**Authors:** Shih-Chuan Chou, Suhas Gondi, Olesya Baker, Arjun K. Venkatesh, Jeremiah D. Schuur

**Affiliations:** 1 Department of Emergency Medicine, Brigham and Women’s Hospital, Boston, Massachusetts; 2 Harvard Medical School, Boston, Massachusetts; 3 Department of Emergency Medicine, Yale School of Medicine, New Haven, Connecticut; 4 Center for Outcomes Research and Evaluation, Yale New Haven Hospital, New Haven, Connecticut

## Abstract

**Importance:**

Insurers have increasingly adopted policies to reduce emergency department (ED) visits that they consider unnecessary. One common approach is to retrospectively deny coverage if the ED discharge diagnosis is determined by the insurer to be nonemergent.

**Objective:**

To characterize ED visits that may be denied coverage if the ED coverage denial policy of a large national insurer, Anthem, Inc, is widely adopted.

**Design, Setting, and Participants:**

A cross-sectional analysis of probability-sampled ED visits from the nationally representative National Hospital Ambulatory Medical Care Survey ED subsample occurring from January 1, 2011, to December 31, 2015, was conducted. Visits by commercially insured patients aged 15 to 64 years were examined. Those with ED discharge diagnoses defined by Anthem’s policy as nonemergent and therefore subject to possible denial of coverage were classified as denial diagnosis visits. The primary presenting symptoms among denial diagnosis visits were identified, and all visits by commercially insured adults presenting with these primary symptoms were classified as denial symptom visits.

**Main Outcomes and Measures:**

Each visit cohort as a weighted proportion of commercially insured adult ED visits. The proportion of each visit cohort that received ED-level care, defined as visits where patients were triaged as urgent or emergent, received 2 or more diagnostic tests, or were admitted or transferred, was also examined.

**Results:**

From 2011 to 2015, 15.7% (95% CI, 15.0%-16.4%) of commercially insured adult ED visits (4440 of 28 304) were denial diagnosis visits (mean [SD] patient age, 36.6 [14.0] years; 2592 [58.7%] female and 2962 [63.5%] white). Among these visits, 39.7% (95% CI, 37.1%-42.3%) received ED-level care: 24.5% (95% CI, 21.7%-27.4%) were initially triaged as urgent or emergent and 26.0% (95% CI, 23.8%-28.3%) received 2 or more diagnostic tests. These denial diagnosis visits shared the same presenting symptoms as 87.9% (95% CI, 87.3%-88.4%) of commercially insured adult ED visits (24 882 of 28 304) (mean [SD] patient age, 38.5 [14.1] years; 14 362 [57.9%] female and 17 483 [68.7%] white). Among these denial symptom visits, 65.1% (95% CI, 63.4%-66.9%) received ED-level care: 43.2% (95% CI, 40.2%-46.4%) were triaged as urgent or emergent, 51.9% (95% CI, 50.0%-53.9%) received 2 or more diagnostic tests, and 9.7% (95% CI, 8.8%-10.6%) were admitted or transferred.

**Conclusions and Relevance:**

Anthem’s nonemergent ED discharge diagnoses were not associated with identification of unnecessary ED visits when assessed from the patient’s perspective. This cost-reduction policy could place many patients who reasonably seek ED care at risk of coverage denial.

## Key Points

**Question:**

If commercial insurers retrospectively deny coverage for emergency department (ED) visits based on diagnoses determined to be nonemergent, what visits will be denied coverage?

**Findings:**

This cross-sectional study found that 1 insurer’s list of nonemergent diagnoses would classify 15.7% of commercially insured adult ED visits for possible coverage denial. However, these visits shared the same presenting symptoms as 87.9% of ED visits, of which 65.1% received emergency-level services.

**Meaning:**

A retrospective diagnosis-based policy is not associated with accurate identification of unnecessary ED visits and could put many commercially insured patients at risk of coverage denial.

## Teaser

This cross-sectional study uses data from the National Hospital Ambulatory Medical Care Survey emergency department (ED) subsample to characterize ED visits that risk denial of coverage by health insurers under retrospective diagnosis-based policies.

## Introduction

Emergency department (ED) visits and payment for ED visits by insurers and patients have grown substantially in the United States over the past several decades.^1,2,3^ This growth has compelled public and commercial payers to pursue strategies to reduce ED care use. One tactic is to apply financial disincentives, such as coverage denial, to ED visits that could presumably be cared for in alternative settings, such as a physician’s office, urgent care center, or retail clinic. These ED visits are often labeled as inappropriate or nonemergent using definitions based on ED discharge diagnoses,^4,5,6^ which are readily available in insurance billing claims. This diagnosis-based approach has not been well studied, but an analysis by Raven et al^7^ using the Billings algorithm demonstrated that nearly 90% of US ED visits had the same presenting symptoms as the ED visits with diagnoses considered primary care–treatable. Their findings indicated that there is no clear link between many presenting symptoms and discharge diagnoses considered primary care–treatable. Because patients make care-seeking decisions based on their symptoms, using a diagnosis-based approach to retrospectively identify inappropriate visits as means of determining coverage may be problematic.

Recently, Anthem, Inc, a large national health insurance company that provides coverage for 1 in 8 Americans,^8^ instituted a policy that will deny coverage and payments for ED visits that it deems unnecessary.^9^ Under this policy, if the final ED diagnosis is among a prespecified list of nonemergent conditions, the insurer will review the ED visit and may deny patient coverage. In 2017, Anthem implemented this policy in Georgia, Missouri, and Kentucky, expanding in 2018 to New Hampshire, Indiana, and Ohio, with expansion to more states under way.^9^ The news media have reported individual cases of patients with Anthem insurance presenting to the ED with concerning symptoms such as abdominal pain or severe headache only to have coverage denied after ED evaluation ruled out emergent conditions.^10,11^ As Anthem remains one of the largest health insurers in the nation, it is important to examine the population that may be affected if other insurers across the nation adopt similar policies of diagnosis-based retroactive coverage denial for ED visits.

Using a national sample of ED visits, we characterized US ED patient visits that may be denied coverage if Anthem’s policy were implemented by all commercial insurers nationally. Our first objective was to examine the ability of this policy to identify inappropriate ED visits by describing ED visits that would be classified as nonemergent under this policy and the proportion of these that received ED-level care. Because patients make care-seeking decisions based on symptoms, our second objective was to describe ED visits with the same presenting symptoms as the nonemergent visits to examine the link between Anthem’s list of nonemergent diagnoses and patient symptoms upon presentation to the ED.

## Methods

### Study Design and Data Set

We performed a cross-sectional analysis of data from January 1, 2011, to December 31, 2015, that were part of the National Hospital Ambulatory Medical Care Survey ED subsample (NHAMCS-ED), a multistage probability sample of US ED visits administered by the National Center for Health Statistics. The NHAMCS-ED uses a 4-stage sampling design: (1) county-level geographic region as primary sampling units, (2) hospitals, (3) emergency service areas, and (4) 100 to 150 patient records from a randomly assigned 4-week period of the survey year. The National Center for Health Statistics excluded federal, military, and Veterans Affairs hospitals. Final samples included from 267 to 408 responding EDs per year. Probability weights and survey design variables were assigned to every visit to allow the calculation of nationally representative estimates and standard errors. Full details of NHAMCS methodology are available online.^12^ This study was exempt from review by the Brigham Heath Institutional Review Board. This study conforms to the Strengthening the Reporting of Observational Studies in Epidemiology (STROBE) reporting guideline for cross-sectional research.^13^

### Participants and Cohort Definitions

To study the potentially eligible commercially insured population, we restricted our analysis to NHAMCS-ED visits by commercially insured adults, defined as patients aged 15 to 64 years with the NHAMCS-ED variable “primary expected source of payment” listed as “private insurance.”

We classified visits into 2 non–mutually exclusive cohorts: (1) denial diagnosis visits and (2) denial symptom visits. The Figure shows the schema used to construct the study cohorts.

**Figure.**
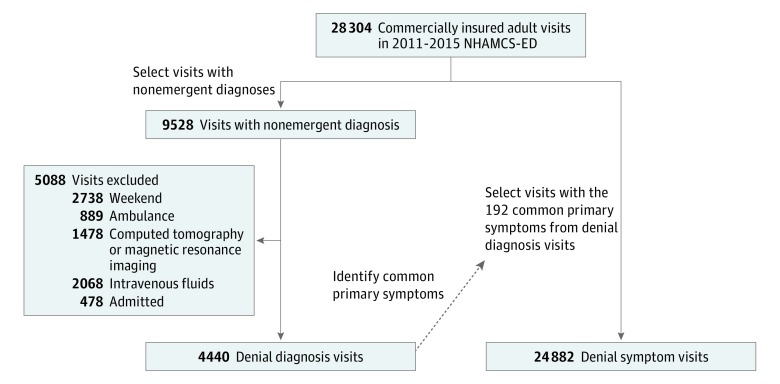
Schema of Study Visit Cohort Definitions We classified visits into 2 non–mutually exclusive cohorts: (1) denial diagnosis visits and (2) denial symptom visits. Sum of the visits for each exclusion criterion exceeds total because some visits have multiple exclusion criteria. NHAMCS-ED indicates National Hospital Ambulatory Medical Care Survey–Emergency Department.

### Denial Diagnosis Visits

To examine the accuracy of Anthem’s policy in identifying unnecessary ED visits, we defined “denial diagnosis visits” as ED visits with at least 1 nonemergent diagnosis specified by Anthem’s denial policy after applying their exclusion criteria. There are 2 publicly published lists of nonemergent diagnoses: the first from Missouri in 2017 (eTable 1 in the Supplement) and the second from Indiana in 2018 (eTable 2 in the Supplement).^9^ We identified visits with nonemergent diagnoses from both lists but based our subsequent analyses on the Indiana diagnosis list, as it is the most recent version of the policy. We then applied the exclusion criteria for Anthem’s coverage denial policy, excluding patients who were younger than 15 years, arrived at the ED during the weekend (defined by Anthem as time between 8 am on a Saturday and 8 am on Monday), arrived via ambulance, received a computed tomographic or magnetic resonance imaging scan, received intravenous fluids, or were admitted to observation or an inpatient ward.^14^ The NHAMCS does not allow identification of several exclusion criteria, including administration of intravenous medications or a patient who was traveling or was located farther than 15 miles from an urgent care center.

### Denial Symptom Visits

The assumption underlying Anthem’s policy is that, prior to ED evaluation, patients should be able to determine that their symptoms were of a nonemergent condition for which ED care is not appropriate. To examine the validity of this assumption, we identified denial symptom visits, defined as all ED visits by commercially insured adults that share the same primary symptoms or reasons for visit with the denial diagnosis visits.

We identified primary symptoms that were commonly associated with diagnoses among the denial diagnosis visits. We first grouped similar diagnoses among the denial diagnosis visits, which are likely to present with similar symptoms, into clinically meaningful categories using the Agency of Healthcare Quality and Research clinical classification software (CCS).^15^ We then removed CCS categories associated with fewer than 5 denial diagnosis visits. For each included CCS category, we identified all unique primary symptoms (using the variable of primary reason for visit) among the denial diagnosis visits with the corresponding CCS category and included any primary symptoms associated with 2 or more denial diagnosis visits.

For example, there were a total of 30 denial diagnosis visits with an ED discharge diagnosis of tonsillitis (CCS 124). Among these tonsillitis visits, there were 8 unique primary symptoms, of which throat soreness was associated with 23 visits, and all other symptoms (such as fever, headache, and earache) were associated with only 1 visit each. Therefore, throat soreness was included as a common primary symptom, but the other symptoms were not. The other presenting symptoms could still be included if they were associated with 2 or more visits for another CCS category.

We performed 2 sensitivity analyses to assess the stability of our findings among denial symptom visits. First, we constructed a denial symptom cohort with a more restrictive definition, including only primary symptoms associated with 5 or more visits within each CCS category. Second, we constructed a denial symptom visit cohort using only 2014 and 2015 NHAMCS-ED data to assess whether there were notable changes after the implementation of the US Affordable Care Act, which included emergency care as 1 of the 10 essential health benefits.

### Statistical Analysis

We report descriptive statistics for each visit cohort. We calculated the proportions of visits in each cohort by patient characteristics (age, sex, race, insurance status, and region), visit characteristics (triage severity and visit timing), ED care received (diagnostic testing and intravenous fluid use), and disposition. For visit timing, we defined evenings as arrival to ED after 5 pm Monday through Friday and before 8 am the next day, weekends as arrival after 8 am on Saturday and before 8 am on Monday (consistent with Anthem’s exclusion criteria), and office hours as the remaining times of the week. We examined the total number of diagnostic services used in each visit, including blood, urine, electrocardiogram, and imaging tests. For disposition, we defined admission as patients admitted to observation or inpatient units.

We also calculated the weighted proportion of visits in each cohort that received “ED-level care,” defined as visits with any of these characteristics: (1) triaged as urgent or emergent, (2) received 2 or more diagnostic tests, or (3) admitted to the hospital or transferred to other facilities.

### Secondary Analyses

To further examine the policy from a patient’s perspective, we performed several secondary analyses. First, we identified the most common primary reasons for visits among denial diagnosis visits and calculated the weighted proportion of visits with each presenting symptom that received ED-level care. Second, we identified the most common reasons for visits among denial symptom visits that were admitted or transferred. Third, we examined the most common reasons for visits among all denial symptom visits and, for each reason for visits, calculated the weighted proportions of visits that were denial diagnosis visits (visits that may be denied coverage under Anthem’s policy) and the weighted proportions that were admitted to the hospital or transferred.

For all analyses, we used SVY procedures in STATA/MP 15 (StataCorp) to incorporate probability weights and survey design variables in generating national estimates for weighted proportions and 95% confidence intervals.

## Results

From 2011 to 2015, NHAMCS-ED included a total of 130 219 ED visits, of which 28 304 were visits by patients aged 15 to 64 years with commercial insurance. These visits represented 21.9% (95% CI, 20.9%-22.9%) of all US ED visits over the study period, accounting for 29.6 million (95% CI, 25.7 million to 33.5 million) ED visits annually. Of these ED visits, 46.6% (95% CI, 45.5%-47.8%) had a nonemergent diagnosis according to the Missouri policy, and 35.5% (95% CI, 32.4%-38.7%) had a nonemergent diagnosis according to the Indiana policy (eTable 3 in the Supplement).

When we applied Anthem’s exclusion criteria to the cohort with the Indiana policy’s nonemergent diagnoses, 15.7% (95% CI 15.0%-16.4%) of adult commercially insured ED visits may be denied coverage (denial diagnosis visits) (4440 of 28 304; mean [SD] patient age, 36.6 [14.0] years; 2592 [58.7%] female and 2962 [63.5%] white), representing 4.6 million (95% CI, 4.1 million to 5.2 million) ED visits annually (Table 1). The most common principal ED discharge diagnoses are shown in eTable 4 in the Supplement. Of the denial diagnosis visits, 24.5% (95% CI, 21.7%-27.4%) were triaged as urgent or emergent and 26.0% (95% CI, 23.8%-28.3%) required 2 or more diagnostic tests. Overall, 39.7% (95% CI, 37.2%-42.3%) of denial diagnosis visits had at least 1 of the characteristics of receiving ED-level care. No patients in the denial diagnosis visit cohort were admitted because admission was an exclusion from Anthem’s coverage denial policy.

**Table 1.** Patient Characteristics, ED Care Provided, and Final Dispositions of Denial Diagnosis Visits and Denial Symptom Visits^a^

Patient Characteristic	Denial Diagnosis Visits (n = 4440)		Denial Symptom Visits (n = 24 882)	
	Unweighted No.	Weighted % (95% CI)^b^	Unweighted No.	Weighted % (95% CI)^b^
Age, y				
15-24	1203	27.7 (25.8-29.7)	5490	22.1 (21.3-22.9)
25-44	1785	38.1 (36.3-40.0)	10 053	40.1 (39.2-40.9)
45-64	1452	34.2 (32.1-36.4)	9339	37.8 (37.0-38.7)
Female	2592	58.7 (56.6-60.7)	14 362	57.9 (56.9-58.9)
Race/ethnicity				
White, non-Hispanic	2962	63.5 (59.4-67.5)	17 483	68.7 (66.2-71.1)
Black, non-Hispanic	874	23.4 (19.9-27.4)	3837	17.6 (15.4-20.1)
Hispanic	434	10.5 (8.8-12.5)	2647	10.8 (9.4-12.3)
Other, non-Hispanic	170	2.5 (2.0-3.2)	915	3.0 (2.4-3.6)
Region				
Northeast	941	19.3 (15.1-24.2)	5173	18.8 (15.0-23.4)
Midwest	1069	23.6 (18.4-29.8)	6320	25.1 (20.1-30.8)
South	1556	39.1 (32.9-45.6)	8164	36.1 (30.9-41.6)
West	874	18.0 (14.1-22.7)	5225	20.0 (16.1-24.6)
Visit timing				
Follow-up visit	240	4.8 (4.0-5.7)	1098	4.2 (3.7-4.7)
Time of day				
Office hours	2199	49.5 (46.3-52.8)	8700	34.7 (33.8-35.6)
Evenings^c^	2241	50.5 (47.2-53.7)	9236	37.4 (36.5-38.4)
Weekends^d^	0	0	6946	27.9 (27.1-28.7)
Was in ED <72 h prior	178	3.7 (2.8-5.0)	996	3.8 (2.9-5.0)
Visit severity				
Triage level				
Urgent or emergent	1134	24.5 (21.7-27.4)	10 870	43.2 (40.2-46.4)
Semiurgent or nonurgent	2036	45.6 (41.8-49.4)	7360	28.9 (26.8-31.2)
Unknown or triage not performed	1270	30.0 (25.3-35.0)	6652	27.8 (24.1-31.9)
Arrived by ambulance	0	0	2480	10.3 (9.8-10.9)
ED care delivered				
Any imaging use	1685	38.9 (36.9-40.8)	12 778	52.0 (50.4-53.6)
Computed tomography or magnetic resonance imaging	0	0	4790	19.6 (18.4-20.8)
Ultrasonography	151	3.3 (2.6-4.3)	1268	5.0 (4.5-5.5)
Total diagnostic services, No.^e^				
0	1741	37.1 (34.7-39.5)	6437	24.9 (23.6-26.2)
1	1604	36.9 (34.7-39.2)	5669	23.2 (22.1-24.2)
2-4	759	18.2 (16.3-20.2)	5517	23.5 (22.3-24.8)
≥5	336	7.8 (6.7-9.2)	7259	28.4 (26.5-30.4)
Intravenous fluids	0	0	7537	30.1 (28.0-32.2)
Disposition				
Admit (observation or inpatient)	0	0	2189	8.2 (7.3-9.2)
Critical care, operating room, or catheterization laboratory	0	0	564	2.0 (1.7-2.4)
Discharge	4160	94.2 (92.9-95.2)	20 874	85.2 (83.9-86.4)
Transfer	13	NC	408	1.4 (1.2-1.7)
Left against medical advice	37	0.8 (0.5-1.3)	372	1.5 (1.3-1.7)
Died upon arrival or in ED	0	0	5	NC
Unknown	230	4.7 (3.6-5.9)	1034	3.7 (3.1-4.4)

Abbreviations: ED, emergency department; NC, not calculated.

^a^ Data are from the National Hospital Ambulatory Medical Care Survey.

^b^ Weighted proportions were not calculated if cells had fewer than 30 records because it creates unstable estimates.

^c^ Evenings defined as 5 pm Monday through Friday to 8 am the next day.

^d^ Weekend defined as 8 am Saturday to 8 am Monday.

^e^ Total diagnostic services include any blood or urine testing, electrocardiogram, or any imaging.

There were 329 unique primary symptoms among the denial diagnosis visits, of which 192 were identified as common primary symptoms (eTable 5 in the Supplement). Among adult commercially insured ED visits, 87.9% (95% CI, 87.3%-88.4%) were denial symptom visits (24 882 of 28 304; mean [SD] patient age, 38.5 [14.1] years; 14 362 [57.9%] female and 17 483 [68.7%] white) (Table 1), of which 43.2% (95% CI, 40.2%-46.4%) were triaged as urgent or emergent, 51.9% (95% CI, 50.0%-53.9%) received 2 or more diagnostic tests, and 9.7% (95% CI, 8.8%-10.6%) were admitted or transferred; 2.0% (95% CI, 1.7%-2.4%) required critical care or immediate intervention in the operating room or cardiac catheterization laboratory. Overall, 65.1% (95% CI, 63.4%-66.9%) of denial symptom visits had at least 1 of these characteristics of receiving ED-level care.

### Sensitivity Analyses

When we restricted the definition of the denial symptom visits to include only primary symptoms with 5 or more visits (94 of the 329 primary symptoms among the denial diagnosis visits) (eTable 5 in the Supplement), 74.7% (95% CI, 73.8%-75.5%) of all commercially insured adult ED visits were included. In this more restrictive visit cohort, the patient characteristics, ED care provided, and final dispositions were not materially different from the primary definition of denial symptom visits. (eTable 6 in the Supplement).

When limiting our analysis to 2014 and 2015 NHAMCS-ED visits, the years after implementation of the Affordable Care Act, 80.0% (95% CI, 78.7%-81.2%) of all included ED visits were denial symptom visits. There were no material changes to patient characteristics, ED care provided, and disposition of these visits from the analysis performed using 2011 to 2015 NHAMCS-ED data (eTable 7 in the Supplement).

### Secondary Analysis

Table 2 shows that, for the most common presenting symptoms among visits that would otherwise be considered nonemergent by the Anthem policy (denial diagnosis visits), a substantial proportion still received ED-level care. Of these presenting symptoms, abdominal pain, chest pain, and headache were also among the most common presenting symptoms of denial symptom visits that led to hospital admission or transfer (Table 3).

**Table 2.** Emergency Department–Level Care Among Denial Diagnosis Visits by 15 Most Frequent Presenting Symptoms

Presenting Symptom^a^	Unweighted No.	Visits Received ED-Level Care^b^	
		Unweighted No.	Weighted % (95% CI)^c^
Total	4440	1749	39.7 (37.2-42.3)
Back pain, ache, soreness, discomfort	241	93	36.0 (28.1-44.7)
Throat soreness	193	54	33.2 (23.0-45.3)
Cough	158	78	43.7 (33.3-54.8)
Skin rash	155	50	35.3 (25.1-47.0)
Ankle pain, ache, soreness, discomfort	119	25	NC
Foot and toe pain, ache, soreness, discomfort	132	29	NC
Toothache	93	14	NC
Leg pain, ache, soreness, discomfort	122	69	50.7 (38.5-62.8)
Low back pain, ache, soreness, discomfort	134	45	32.9 (24.3-42.8)
Abdominal pain, cramps, spasms, none otherwise specified	105	99	96.2 (89.7-98.7)
Earache, pain	99	14	NC
Knee pain, ache, soreness, discomfort	99	22	NC
Shoulder pain, ache, soreness, discomfort	100	29	NC
Headache, pain in head	77	41	55.8 (40.5-70.1)
Chest pain	69	53	75.5 (59.9-86.5)

Abbreviations: ED, emergency department; NC, not calculated.

^a^ Presenting symptoms were classified using the reason for visit codes.

^b^ We defined ED-level care as a visit that was triaged as urgent or emergent or received 2 or more diagnostic tests. Of note, no patients in the denial diagnosis visit cohort were admitted because admission was an criterion for exclusion from Anthem's coverage denial policy.

^c^ Weighted proportions for cells with fewer than 30 numerator observations were not calculated because it produces unstable estimates.

**Table 3.** Top 15 Presenting Symptoms Among the Denial Symptom Visits That Were Admitted to Hospital or Transferred

Presenting Symptom^a^	Denial Symptom Visits Admitted or Transferred (n = 2189)	
	Unweighted No.	Weighted % (95% CI)^b^
Chest pain	420	21.4 (19.0-24.0)
Abdominal pain, cramps, spasms, none otherwise specified	312	15.3 (13.5-17.4)
Shortness of breath	155	6.8 (5.4-8.4)
Nausea	59	2.5 (1.8-3.4)
Other symptoms or problems related to psychological or mental disorder	51	2.3 (1.6-3.2)
Fever	52	2.1 (1.5-3.0)
Lower abdominal pain, cramps, spasms	50	2.0 (1.5-2.8)
Vertigo or dizziness	56	2.0 (1.5-2.7)
Upper abdominal pain, cramps, spasms	35	2.0 (1.3-2.9)
Side pain, flank pain	38	1.9 (1.3-2.8)
Headache, pain in head	45	1.8 (1.3-2.6)
Chest discomfort, pressure, tightness	30	1.7 (1.1-2.6)
Vomiting	44	1.5 (1.0-2.3)
Medical counseling, none otherwise specified	29	NC
General weakness	35	1.4 (0.9-2.1)

Abbreviation: NC, not calculated.

^a^ Presenting symptoms were classified using the reason for visit codes.

^b^ Weighted proportions for cells with fewer than 30 numerator observations were not calculated because it produces unstable estimates.

Among visits for the most common presenting symptoms in denial symptom visits, many presenting symptoms with high rates of admission or transfer also had a meaningful proportion of ED visits that could potentially be denied coverage (denial diagnosis visits) (Table 4). For example, among denial symptom visits with abdominal pain, 15.9% (95% CI, 13.6%-18.6%) required admission or hospital transfer but 4.3% (95% CI, 3.3%-5.6%) may be considered nonemergent and potentially denied coverage.

**Table 4.** Proportion of Denial Symptom Visits Resulting in Potential Coverage Denial or in Admission or Transfer by 15 Most Frequent Presenting Symptoms

Presenting Symptom^a^	Total Unweighted No.	Potential Coverage Denial^b^		Admission or Transfer	
		Unweighted No.	Weighted % (95% CI)^c^	Unweighted No.	Weighted % (95% CI)^c^
Total	24 882	4256	17.1 (16.3-17.9)	2597	9.7 (8.8-10.6)
Abdominal pain, cramps, spasms, none otherwise specified	2086	105	4.3 (3.3-5.6)	347	15.9 (13.6-18.6)
Chest pain	1684	69	3.9 (2.9-5.3)	475	26.5 (23.2-30.0)
Headache, pain in head	1041	77	7.7 (5.7-10.2)	58	5.0 (3.7-6.8)
Back pain, ache, soreness, discomfort	888	241	29.0 (24.8-33.6)	36	3.3 (2.2-4.9)
Side pain, flank pain	590	25	NC	41	6.6 (4.5-9.6)
Vertigo or dizziness	576	27	NC	61	8.3 (6.1-11.1)
Shortness of breath	564	37	6.7 (4.5-9.9)	171	26.0 (20.8-32.0)
Cough	544	158	27.4 (21.7-33.9)	19	NC
Laceration or cut of upper extremity	497	19		11	NC
Nausea	470	30	6.0 (3.4-10.3)	66	13.0 (9.6-17.4)
Low back pain, ache, soreness, discomfort	439	134	25.5 (20.4-31.4)	11	NC
Vomiting	436	20	NC	55	9.6 (6.5-14.1)
Throat soreness	419	193	47.0 (39.3-55.0)	6	NC
Injury, other and unspecified of head, neck, and face	405	50	9.7 (6.6-14.1)	28	NC
Neck pain, ache, soreness, discomfort	385	45	10.8 (7.7-14.9)	12	NC

Abbreviation: NC, not calculated.

^a^ Presenting symptoms were classified using the reason for visit codes.

^b^ These denial symptom visits were also denial diagnosis visits, that is, visits with nonemergent diagnoses that may be denied coverage according to Anthem’s policy.

^c^ Weighted proportions for cells with fewer than 30 numerator observations were not calculated because it produces unstable estimates.

## Discussion

Amidst rising health care costs, public and commercial insurers are adopting policies to limit their payments for emergency care. One approach recently implemented by a major commercial insurer, Anthem, is to disincentivize unnecessary ED visits by denying coverage and payments for visits with nonemergent ED discharge diagnoses. Our results demonstrate the inaccuracy of such a policy in identifying unnecessary ED visits. Furthermore, patients cannot reliably avoid coverage denial as most presenting symptoms could potentially lead to a nonemergent diagnosis.

The main limitation of retrospectively judging the necessity of ED care is that the determination is based on information not available to patients prior to the medical evaluation. When patients become acutely ill, they must decide whether to seek care (and, if so, when and where) based not on a diagnosis but on the symptoms they are experiencing. The link between presenting symptoms, ED care required, and final diagnoses is often uncertain. While clinicians possess the appropriate training to elucidate the care patients need, as our results show, a diagnosis-based algorithm cannot capture the complexity of these decisions.

We found that if Anthem’s policy was adopted by all other commercial insurers, nearly 1 in 6 ED visits by commercially insured adults would have a nonemergent ED discharge diagnosis and could be denied coverage. Moreover, in up to 9 of 10 adult commercially insured ED visits, patients presented with the same primary symptoms as the visits that resulted in nonemergent diagnoses that could be denied coverage. Even among patients with potentially life-threatening symptoms such as chest pain, who would likely be instructed to seek emergency care if they consulted outpatient clinicians, up to 4% may be denied coverage and possibly receive an uncovered medical bill. This result is consistent with the study by Raven et al,^7^ who found the presenting symptoms from ED visits with “primary care-treatable” diagnoses, as defined by the Billings algorithm, were present in nearly 9 of 10 ED visits. These findings illustrate that the link between presenting symptoms and final ED diagnoses are often not straightforward. From the patients’ perspective, if similar retrospective coverage denial policies were implemented widely, up to 90% of patients have symptoms that may lead to nonemergent ED diagnoses. These patients would, therefore, need to consider the possibility of coverage denial before seeking ED care.

Furthermore, nearly 40% of visits that had nonemergent diagnoses and could be denied coverage received substantial ED care, including being triaged as urgent or emergent and receiving multiple diagnostic tests. Even triage nurses, the most experienced ED nurses, considered nearly one-quarter of visits with nonemergent ED discharge diagnoses urgent or emergent prior to a full clinical evaluation. These results demonstrate the substantial discrepancy between what may be considered an unnecessary ED visit based on ED discharge diagnoses compared with a prospective assessment based on patients’ presentation.

The absence of reliable information on patient presentation, such as presenting symptoms or reasons for visit, in administrative billing data remains a key barrier in examining the appropriateness of ED care. This is highlighted by a new National Quality Forum project that aims to develop systems for chief complaint–based quality of emergency care.^16^ Therefore, insurers implementing similar policies of retrospective denial based on billing data would need to devote substantial resources to audit and review the medical records of up to 1 in 6 adult ED visits. A recent Senate investigation of Anthem’s policy found that less than half of the visits that were initially flagged and reviewed were ultimately denied coverage, confirming the inaccuracy and inefficiency of this approach.^17^

How a retrospective coverage denial policy, such as that implemented by Anthem, would influence ED utilization and patient outcomes remains to be studied. Existing evidence evaluating the effects of ED cost sharing shows that even modest copayments of $25 to $35 would lead to decreases in both low-severity and high-severity ED visits.^18,19^ Cost sharing in the form of high-deductible health plans have also been associated with decreased low-severity ED visits.^20^ But among populations of lower socioeconomic status, high deductibles also reduce high-severity ED visits.^20^ In contrast to copayments or high deductibles, diagnosis-based coverage denial poses a financial disincentive that further adds an element of uncertainty. As a result, patients with acute illnesses are put in a difficult position of weighing the risk of delayed treatment for severe disease vs an uncovered medical bill.

Three decades ago, managed care organizations and insurers deterred patients from emergency care through similar strategies of coverage denial and preauthorization requirements.^21^ They used triage algorithms, which were unable to exclude the presence of severe disease with adequate sensitivity, leading to adverse events and even deaths.^21,22^ In response, states and the federal government passed laws requiring insurers to cover emergency care based on the “prudent layperson standard.” The standard defines “emergency medical conditions” as “acute symptoms of sufficient severity (including severe pain) such that a prudent layperson, who possesses an average knowledge of health and medicine, could reasonably expect the absence of immediate medical attention to result in [serious deterioration of health].”^23^ In 1992, Maryland passed the first prudent layperson law. Other states soon followed, and in 1997 it became federal law in the Balanced Budget Act. In 2010, the Affordable Care Act included this standard for emergency care coverage as 1 of the 10 essential health benefits.

Anthem’s policy of retroactive coverage denial based on discharge diagnosis has led to a congressional investigation by Sen Claire McCaskill (D, Missouri) for potential violation of the prudent layperson standard.^17^ This investigation found that, while 10% to 20% of ED visits were reviewed by Anthem for denial, only 4% to 7% of ED visits were ultimately denied.^17^ While the proportion denied is small, it is not clear if this will expand over time to additional states or be adopted by other insurers. Both the American College of Emergency Physicians and the American Medical Association have expressed similar concerns about this policy,^24,25^ particularly given Anthem’s prominence as one of the nation’s largest health insurers. If retrospective denial policies are widely adopted, they would place undue financial stress on patients with acute illness and could increase barriers to timely emergency care, particularly to those least able to pay.

### Limitations

Our study is bound by the limitations intrinsic to a cross-sectional national survey, including potential misclassification of presenting symptoms, ED care received, or discharge diagnoses.^26^ Two additional limitations warrant mention. First, 2 of the exclusion criteria published by Anthem could not be operationalized in NHAMCS-ED. We could not exclude visits based on intravenous medication use as NHAMCS-ED does not contain information on the route of medication administration. Based on prior studies on use of peripheral intravenous catheter in the ED, we anticipate that accounting for patients receiving intravenous medications would have reduced the proportion of adult commercially insured patients at risk of coverage denial by approximately 1%, as there is substantial overlap with patients receiving intravenous fluids and other exclusion criteria.^27,28^ Also, we could not determine whether visits occurred while the patient was traveling or was outside of a 15-mile range from an urgent care center. These visits likely account for a small proportion of ED visits, particularly as the number of urgent care centers has increased dramatically, with 6707 centers across the United States by 2015.^29^ Second, the primary symptoms from denial diagnosis visits that we used to identify denial symptom visits may be overly inclusive, resulting in the inclusion of uncommon symptoms among denial symptom visits. In our primary analysis we included only primary symptoms that occurred in 2 or more visits of the same diagnosis category. We further performed a sensitivity analysis restricting to primary symptoms present in 5 or more visits of the same diagnosis category. We found no substantial changes in the patient characteristics, ED care delivered, and final dispositions of denial symptom visits between the primary and sensitivity analysis, suggesting that our approach is not overly inclusive.

## Conclusions

One in 6 ED visits by commercially insured adults could be denied coverage if a cost-reduction policy recently implemented by a large commercial insurer is widely adopted. However, the policy cannot accurately identify unnecessary visits, as up to 40% of the visits that were considered nonemergent were likely appropriate ED visits. Furthermore, these visits presented with the same spectrum of symptoms as nearly 9 of 10 ED visits. This cost-reduction policy could place many patients who reasonably seek ED care at risk of coverage denial.

## Notes

Supplement. **eTable 1.** List of Nonemergent Diagnosis per Missouri Policy **eTable 2.** List of Nonemergent Diagnosis per Indiana Policy **eTable 3.** Characteristics of Commercially-Insured Adult ED Patients With “Non-Emergent” ED Discharge Diagnoses, Comparing Missouri vs Indiana Policy **eTable 4.** Frequencies and Proportions of Top 20 Diagnoses Among Denial Diagnosis Visits **eTable 5.** Complete List of 329 Reasons for Visit Associated With Denial Diagnosis Visits **eTable 6.** Denial Symptom Visits as Defined by Primary Symptoms Present in ≥2 or ≥5 Denial Diagnosis Visits **eTable 7.** Denial Symptom Visits, by Data Years
