# Supplementary material for: Analysis of a Commercial Insurance Policy to Deny Coverage for Emergency Department Visits With Nonemergent Diagnoses
Source: JAMA Netw Open. 2018 Oct 19;1(6):e183731. doi: 10.1001/jamanetworkopen.2018.3731 (PMC6324426; doi:10.1001/jamanetworkopen.2018.3731)
Supplement: Supplement. — eTable 1. List of Nonemergent Diagnosis per Missouri Policy eTable 2. List of Nonemergent Diagnosis per Indiana Policy eTable 3. Characteristics of Commercially-Insured Adult ED Patients With “Non-Emergent” ED Discharge Diagnoses, Comparing Missouri vs Indiana Policy eTable 4. Frequencies and Proportions of Top 20 Diagnoses Among Denial Diagnosis Visits eTable 5. Complete List of 329 Reasons for Visit Associated With Denial Diagnosis Visits eTable 6. Denial Symptom Visits as Defined by Primary Symptoms Present in ≥2 or ≥5 Denial Diagnosis Visits eTable 7. Denial Symptom Visits, by Data Years [file jamanetwopen-1-e183731-s001.pdf]

Supplementary Online Content

Chou S-C, Gondi S, Baker O, Venkatesh AK, Schuur JD. Analysis of a commercial insurance policy to deny coverage for emergency department visits with nonemergent diagnoses. *JAMA Netw Open*. 2018;1(6):e183731. doi:10.1001/jamanetworkopen.2018.3731

eTable 1. List of Nonemergent Diagnosis per Missouri Policy

eTable 2. List of Nonemergent Diagnosis per Indiana Policy

eTable 3. Characteristics of Commercially-Insured Adult ED Patients With "Non-Emergent" ED Discharge Diagnoses, Comparing Missouri vs Indiana Policy

eTable 4. Frequencies and Proportions of Top 20 Diagnoses Among Denial Diagnosis Visits

eTable 5. Complete list of 329 Reasons for Visit Associated With Denial Diagnosis Visits

eTable 6. Denial Symptom Visits as Defined by Primary Symptoms Present in ≥2 or ≥5 Denial Diagnosis Visits

eTable 7. Denial Symptom Visits, by Data Years

This supplementary material has been provided by the authors to give readers additional information about their work.

© 2018 Chou S-C et al. *JAMA Network Open.*

eTable 1. List of Nonemergent Diagnosis per Missouri Policy

Diagnosis	ICD-9 Code
1St Deg Burn Multiple Finger	94413
1St Deg Burn Wrist	94417
Abrasion Head	9100
Absence Of Menstruation	6260
Acute Apical Periodontitis	5224
Acute Atopic Conjunctivitis	37205
Acute Ethmoidal Sinusitis	4612
Acute Follicular Conjunctivitis	37202
Acute Frontal Sinusitis	4611
Acute Infection Extern Ear NEC	38013
Acute Maxillary Sinusitis	4610
Acute Nonsupprative Otitis Med NOS	38100
Acute Serous Otitis Media	38101
Acute Sphenoidal Sinusitis	4613
Acute Supprative Otitis Media In diseases classified elsewhere	38202
Acute Supprative Otitis Media with Spontaneous Rupture of Ear Drum	38201
Acute Supprative Otitis Media NOS	38200
Acne NEC	7061
Acute Bronchitis	4660
Acute Conjunctivitis NOS	37200
Acute Cystitis	5950
Acute Gastritis without Hemorrhage	53500
Acute Gonococcal Infection Lower Genitourinary Tract	0980
Acute Infection Of Pinna	38011
Acute Laryngitis without Obstruction	46400
Acute Laryngopharyngitis	4650
Acute Nasopharyngitis	460
Acute Pharyngitis	462
Acute Sinusitis NOS	4619
Acute Swimmers Ear	38012
Acute Tonsillitis	463
Acute Uri Multiple Sites NEC	4658
Allergic Rhinitis Due to Animal Hair & Dander	4772
Allergic Rhinitis Due To Food	4771
Allergic Rhinitis NEC	4778
Allergic Rhinitis NOS	4779
Allergy, Unspecified	9953
Angular Blepharoconjunctivitis	37221
Anxiety State NOS	30000
Arthropathy NOS-Unspecified	71690
Back Contusion	92231
Backache NOS	7245
Blepharoconjunctivit NOS	37220
Blood Typing Encounter (Begin 2005)	V7286
Bronchitis NOS	490
Candidal Vulvovaginitis	1121
Candidiasis Site NOS	1129
Cellulitis Of Arm	6823
Cellulitis Of Buttock	6825
Cellulitis Of Leg	6826
Cellulitis Of Trunk	6822
Cervicalgia	7231
Cervicitis	6160
Chalazion	3732
Chancroid	0990
Chemical Dermatitis NEC	6924

© 2018 Chou S-C et al. *JAMA Network Open.*

Diagnosis	ICD-9 Code
Chronic Allergic Conjunctivitis NEC	37214
Chronic Conjunctivitis NOS	37210
Chronic Ethmoidal Sinusitis	4732
Chronic Fatigue Syndrome (Begin 1998)	78071
Chronic Follicular Conjunctivitis	37212
Chronic Frontal Sinusitis	4731
Chronic Maxillary Sinusitis	4730
Chronic Serous Otitis Media Simple/NOS	38110
Chronic Sphenoidal Sinusitis	4733
Chronic Suppurative Otitis Media NOS	3823
Chronic Rhinitis	4720
Chronic Sinusitis NEC	4738
Chronic Sinusitis NOS	4739
Condyloma Accuminatum (Begin 1993)	07811
Conjunctiva Disorder NOS	3729
Conjunctival Hemorrhage	37272
Conjunctivitis NEC	37239
Conjunctivitis NOS	37230
Constipation, Unspecified	56400
Contact Blepharoconjunctvitis	37222
Contusion Face/Scalp/Neck	920
Contusion Of Ankle	92421
Contusion Of Chest Wall	9221
Contusion Of Elbow	92311
Contusion Of Finger	9233
Contusion Of Foot	92420
Contusion Of Forearm	92310
Contusion Of Hand(S)	92320
Contusion Of Knee	92411
Contusion Of Lower Leg	92410
Contusion Of Upper Arm	92303
Contusion Of Wrist	92321
Cough	7862
Coxsackie Virus Inf NOS	0792
Cramp In Limb	72982
Cystitis NOS	5959
Dental Caries, Unspecified	52100
Dental Disorder NEC	5258
Dental Disorder NOS	5259
Dental Examination	V722
Dermatitis Due To Plant	6926
Dermatitis NOS	6929
Dermatographic Urticaria	7083
Dermatophytosis Scalp/Beard	1100
Dermatophytosis Of Body	1105
Dermatophytosis Of Foot	1104
Dermatophytosis Of Groin	1103
Dermatophytosis Site NEC	1108
Dermatophytosis Site NOS	1109
Detergent Dermatitis	6920
Diaper Or Napkin Rash	6910
Diarrhea	78791
Dis Of Conjunct Due To Viruses (Begin 1993)	07799
Drug Dermatitis NOS	6930
Dysmenorrhea	6253
Dysuria	7881
Edema	7823

© 2018 Chou S-C et al. *JAMA Network Open.*

Diagnosis	ICD-9 Code
Elbow/Forearm/Wrist Injury NOS	9593
Encounter Change Or Remove Surgical Wound Dressing	V5831
Encounter For Removal Of Sutures	V5832
Encounter-Change Or Remove Nonsurgical Wound Dressing	V5830
Enlargement Lymph Nodes	7856
Enthesopathy, Site NOS	72690
Esophageal Reflux	53081
Exam Ears & Hearing NEC (Begin 2006)	V7219
External Hemorrhoid without Complication	4553
Eye & Vision Examination	V720
Fem Genital Symptoms NOS	6259
Flu W Resp Manifest NEC	4871
Follow-Up Exam NEC	V6759
Follow-Up Exam NOS	V679
Food Poisoning NOS	0059
Foreign Body Skin	7094
Foreign Body In Ear	931
Gastroenteritis	0088
General Medical Exam NEC	V708
Genital Herpes NEC	05419
Genital Herpes NOS	05410
Gingivitis/Periodontitis Disorder NOS	5239
Gout NOS	2749
Group B Streptococcal Carrier	V0251
Gastritis/Duodenitis NOS without Hemorrhage	53550
Hand- Foot & Mouth Dis	0743
Hand Injury NOS	9594
Health Exam-Group Survey	V705
Hearing Conservation/Treatment (Begin 2007)	V7212
Hearing Exam-Fail Screen (Begin 2006)	V7211
Hepatitis B Carrier	V0261
Hernia NOS	5539
Herpangina	0740
Herpes Simplex NOS	0549
Herpes Zoster NOS	0539
Herpetic Gingivostomatitis	0542
Hip & Thigh Injury NOS	9596
Hordeolum Externum	37311
Hypertension	4019
Hypopotassemia	2768
Impacted Cerumen	3804
Impetigo	684
Infectious Otitis Externa NOS	38010
Infectious Mononucleosis	075
Infestation NOS	1349
Ingrowing Nail	7030
Insect Bite NEC-Infected	9195
Insomnia	78052
Issue Repeat Prescript	V681
Joint Pain-Ankle	71947
Joint Pain-Forearm	71943
Joint Pain-L/Leg	71946
Joint Pain-Shoulder	71941
Joint Pain-Unspecified	71940
Joint Pain-Up/Arm	71942
Lower Leg Injury NOS	9597
Lumbago	7242

© 2018 Chou S-C et al. *JAMA Network Open.*

Diagnosis	ICD-9 Code
Malaise And Fatigue NEC	78079
Male Genital Dis NOS	6089
Med Exam NEC-Admin Purpose	V703
Menstrual Disorder	6268
Menstrual Disorder NOS	6269
Mononeuritis Arm NOS	3549
Mononeuritis NOS	3559
Mucopurulent Conjunctivitis NEC	37203
Myalgia And Myositis NOS	7291
Nausea Alone	78702
Noise Effect-Ear/NOS	38810
Noninfectious Gastroenteritis NEC	5589
Noninflammatory Disorder of Vagina NEC	6238
Nonspecific Skin Eruption NEC	7821
Nonsupprative Otitis Media NOS	3814
Oil & Grease Dermatitis	6921
Oral Aphthae	5282
Osteoarthrosis NOS-L/Leg	71596
Osteoarthrosis NOS-Unspecified	71590
Otalgia NOS	38870
Other Spec Exam (Begin 1993)	V7285
Other Spec Pre-Op Exam (Begin 1993)	V7283
Other Acute Sinusitis	4618
Other Atopic Dermatitis	6918
Other Back Symptoms	7248
Other Diseases Of Nasal Cavity And Sinuses	47819
Other General Symptoms	78099
Other Injury Of Other Sites Of Trunk	95919
Other Psoriasis	6961
Other Spec Viral Warts (Begin 1993)	07819
Otitis Media NOS	3829
Otorrhea NOS	38860
Ovarian Cyst	6202
Pain In Limb	7295
Painful Respiration	78652
Pap Smear Confirmation (Begin 2004)	V7232
Parasitic Conjunctivitis	37215
Patellar Tendinitis	72664
Pediculosis NOS	1329
Periapical Abscess	5225
Phthisis Pubis	1322
Pityriasis Rosea	6963
Pityriasis Versicolor	1110
Plantar Wart (Begin 2008)	07812
Pregnant State- Incidental	V222
Pregnancy Test Negative (Begin 2004)	V7241
Pregnancy Test Unconfirm (Begin 2004)	V7240
Pregnancy Test-Positive (Begin 2005)	V7242
Premenstrual Tension	6254
Pre-Op Cardiovascular Exam (Begin 1993)	V7281
Preop Exam Unspecified	V7284
Pre-Op Respiratory Exam (Begin 1993)	V7282
Pruritic Conditions NEC	6988
Pruritic Disorder NOS	6989
Pseudomembranous Conjunctivitis	37204
Pure Hypercholesterolem	2720
Radiological Exam NEC	V725

© 2018 Chou S-C et al. *JAMA Network Open.*

Diagnosis	ICD-9 Code
Referral-No Exam/Treat	V6881
Rhinitis Due To Pollen	4770
Routine Child Health Exam	V202
Routine Gyn Examination (Begin 2004)	V7231
Routine Medical Exam	V700
Scabies	1330
Sciatica	7243
Screen For Hypertension	V811
Screen-Diabetes Mellitus	V771
Screening Eye Cond NEC	V802
Screening For Lipoid Disorders	V7791
Screening For Obesity	V778
Screening-Pulmonary Tb	V741
Sebaceous Cyst	7062
Sebaceous Gland Dis NOS	7069
Seborrheic Dermatitis NOS (Begin 1995)	69010
Serous Conjunctivitis	37201
Shoulder/Upper Arm Injury NOS	9592
Simple Chronic Conjunctivitis	37211
Skin Disorder NOS	7099
Skin Disorders NEC	7098
Skin Sensation Disturb	7820
Solar Dermatitis NOS	69270
Solvent Dermatitis	6922
Sprain Elbow/Forearm NOS	8419
Sprain Lumbar Region	8472
Sprain NEC	8488
Sprain Of Ankle NOS	84500
Sprain Of Back NOS	8479
Sprain Of Foot NOS	84510
Sprain Of Hand NOS	84210
Sprain Of Wrist NOS	84200
Sprain Shoulder/Arm NOS	8409
Sprain Thoracic Region	8471
Strep Sore Throat	0340
Streptococcus Carrier NEC	V0252
Sunburn	69271
Suppurative Otitis Media NOS	3824
Swelling In Head & Neck	7842
Swelling Of Limb	72981
Throat Pain	7841
Thrush	1120
Tinnitus NOS	38830
TMJ Disorders NOS	52460
Topical Food Dermatitis	6925
Topical Med Dermatitis	6923
Torticollis NOS	7235
Trichomonal Vaginitis	13101
Trichomoniasis NOS	1319
Urethral Discharge	7887
Urethritis NOS	59780
Urin Tract Infection NOS	5990
Urinary Frequency	78841
Urinary Tract Dis NOS	5999
Urticaria NEC	7088
Urticaria NOS	7089
Vaccin For Diphtheria	V035

© 2018 Chou S-C et al. *JAMA Network Open.*

Diagnosis	ICD-9 Code
Vaginitis NOS	61610
Varicella Uncomplicated	0529
Venereal Disease NOS	0999
Vernal Conjunctivitis	37213
Viral Exanthemata NOS	0579
Viral Hep Carrier NOS	V0260
Viral Infection NOS	07999
Viral Warts Unspec (Begin 1993)	07810
Vomiting Alone	78703

Abbreviations: ICD, international classification of disease; NEC, none elsewhere classified; NOS, none otherwise specified
Note: ICD-9 code with implied decimal point after 3rd digit from the left.

© 2018 Chou S-C et al. *JAMA Network Open.*

eTable 2. List of Nonemergent Diagnosis per Indiana Policy

Diagnosis	ICD-9 Code
Abrasion	91*0
Acute Pharyngitis	462
Acute Serous Otitis Media	38101
Acne Vulgaris	7061
Acute Atopic Conjunctivitis Unspecified Eye	37205
Acute Bronchitis Due to Spec Organisms	4660
Acute Bronchitis Unspecified	4660
Acute Conjunctivitis	37200
Acute Laryngitis	46400
Acute Nasopharyngitis Common Cold	460
Acute Pharyngitis Unspecified	462
Acute Serous Otitis Media Bilateral	38101
Acute Streptococcal Tonsillitis unspecified	0340
Acute Tonsillitis Unspecified	463
Acute Up Respiratory Infection Unspecified	4658
Acute Vaginitis	61610
Allergic Contact Dermatitis	692*
Allergic Rhinitis	477*
Allergic Urticaria	708*
Allergy Unspecified Initial Encounter	9953
Amenorrhea Unspecified	6260
Anogenital Venereal Warts	07811
Arthropathy Unspecified	71690
Atopic Conjunctivitis Eye	37205
Bullous Impetigo	684
Candidal Stomatitis	1120
Cellulitis	6823 6822 6825 6826 38010
Chalazion Right Upper Eyelid	3732
Chronic Serous Otitis Media	38110
Conjunctivitis (Pink Eye)	3720* 3721* 3722* 3723* 077*
Contact Dermatitis	692*
Contusion Ankle	92421
Contusion Upper Arm	92303
Contusion Ear	920
Contusion Foot	92420
Contusion Knee	92411
Contusion Lower Leg	92410
Contusion Neck	920
Contusion Of Nose	920
Contusion Scalp	920
Contusion Thigh	92400
Contusion Toes	9243

© 2018 Chou S-C et al. *JAMA Network Open.*

Diagnosis	ICD-9 Code
Cough	7862
Dermatitis Unspecified	692*
Dermatophytosis Unspecified	1109
Disorder Teeth Supporting Structure Unspecified	5258 5259
Encounter Change/Removal Nonsurgical Wound Dressing	V5830
Encounter Change/Removal Surg Wound Dressing	V5831
Encounter Follow-up Exam After Completion Treatment Not Malignant neoplasm	V6759
Encounter For Issue Repeat Prescription	V681
Encounter For Other General Examination	V708
Encounter Gen Adult Exam without Abnormal Find	V700
Encounter Pregnancy Test Result Negative/Positive	V724*
Encounter Routine Child Health Exam without Abnormal Finding	V202
Encounter Exam Bp without Abnormal Find	V7285
Encounter For Immunization	V035
Encounter For Removal Of Sutures	V5832
Encounter Other Admin Examinations	V703
Encounter Other Spec Special Exams	V725
Encounter Screen For Other Disorder	V778
Enteroviral Vesicular Pharyngitis	0740
Enthesopathy Unspecified	72690
Essential Primary Hypertension	4019
Fibromyalgia	7291
Follicular Disorder Unspecified	7069
General Skin Eruption Due to Rx Taken Internally	6930
Hordeolum Externum Eyelid	37311
Impacted Cerumen	3804
Impetigo Unspecified	684
Ingrowing Nail	7030
Laceration without Fb Knee Or Lower Leg Initial/Recurrent	891*
Low Back Pain	7242
Miliaria Rubra	7051
Myalgia	7291
Nasal Congestion	47819
Nausea	78702
Noninfective Ge & Colitis Unspecified	5589
Nonsuppurative Otitis Media	38100
Other Mucopurulnt Conjunctivit Bilateral	37203
Other Skin Change Chrn Exps Nonionz Rad	69270
Other Specified Condition of Female Genital Organ Menstrual Cycle	6259
Other Specified Disorder of Teeth Supporting Structure	5258
Other Specified Noninfective Gastroenteritis & Colitis	5589
Other Specified Noninflammatory Disorder Vagina	6238
Other Allergy Initial Encounter	9953
Other Fatigue	78079

© 2018 Chou S-C et al. *JAMA Network Open.*

Diagnosis	ICD-9 Code
Other Seasonal Allergic Rhinitis	4778
Other Spec Soft Tissue Disorders	72981
Other Urticaria	708*
Other Viral Warts	07819
Otitis Externa	38013
Otitis Media	38202
Otitis Media Unspecified Bilateral	3829
Pain In Extremities (All)	7295 7194*
Pain In Throat	7841
Pityriasis Rosea	6963
Plantar Wart	07812
Pregnant State Incidental	V222
Primary Insomnia	78052
Proc&Tx Not Carried Out Other Reasons	V64*
Pruritus Unspecified	6989
Puncture Wound without Foreign Body Leg	891*
Rash Other Nonspecific Skin Eruption	7821
Recurrent Oral Aphthae	5282
Scabies	1330
Sebaceous Cyst	7062
Serous Conjunctivitis	37201
Sprain /Strain Or Unspecified Injury In Extremities	840* 841* 842* 845* 848* 8471 8472 8479
Streptococcal Pharyngitis	0340
Sunburn Of First Degree	69271
Sunburn Unspecified	69271
Swimmers Ear Rt/Lt/Bilateral	38012
Temporomandibular Joint Disorder Unspecified	52460
Tinea Infections	110*
Unspecified Open Wound Lt/Rt Lower Leg	891*
Unspecified Viral Inf Skin & Mucus Membrane	0579
Unspecified Conjunctivitis	37230
Unspecified Injury	959*
Unspecified Sprain	848.9, 848.9
Upper Respiratory Infection	4658
UTI Site Not Specified	5990
Vaginitis	6161*
Varicella Without Complication	0529
Viral Conjunctivitis Unspecified	07799
Viral Infection Unspecified	0088
Viral Wart Unspecified	07810

Abbreviations: ICD, international classification of disease
Note: Available diagnosis list contains only diagnosis description. Therefore, association with ICD-9 codes were assigned by the authors, with reference to the Missouri policy. ICD-9 code with implied decimal point after 3rd digit from the left. '*' can be replaced by any digit.

© 2018 Chou S-C et al. *JAMA Network Open.*

eTable 3. Characteristics of Commercially-Insured Adult ED Patients with "non-Emergent" ED Discharge Diagnoses, Comparing Missouri vs Indiana Policy

	2011-2015 National Hospital Ambulatory Medical Care Survey ED Subsample (n=28,304)					
	Missouri Policy Visits (n=13,006)			Indiana Policy Visits (n=9.528)		
	Unweighted Count	Weighted % (95% CI)		Unweighted Count	Weighted % (95% CI)	
**Demographics**						
**age (category)**						
15-24 years old	3,009	23.5	(22.5-24.6)	2,358	24.9	(23.7-26.1)
25-44 years old	5,394	40.6	(39.5-41.7)	3,781	38.6	(37.1-40.1)
45-64 years old	4,603	35.9	(34.8-37.1)	3,389	36.5	(35.0-38.1)
**Female**	7,815	60.5	(59.1-61.8)	5,624	59.9	(58.5-61.3)
**Race**						
White, Non-Hispanic	8,908	66.0	(62.9-69.0)	6,486	65.7	(62.4-68.8)
Black, Non-Hispanic	2,281	20.3	(17.6-23.3)	1,686	21.0	(18.1-24.4)
Hispanic	1,354	10.8	(9.3-12.4)	996	10.6	(9.1-12.2)
Other, Non-Hispanic	463	3.0	(2.4-3.7)	360	2.7	(2.2-3.4)
**Region**						
Northeast	2,614	17.7	(14.1-22.1)	1,962	18.1	(14.4-22.4)
Midwest	3,198	24.5	(19.4-30.5)	2,301	24.5	(19.3-30.6)
South	4,564	38.4	(32.8-44.2)	3,366	39.0	(33.3-45.0)
West	2,630	19.4	(15.5-24.0)	1,899	18.4	(14.6-23.0)
**Visit timing**						
**Follow-up Visit**	575	4.0	(3.5-4.6)	461	4.3	(3.7-5.0)
**Time of day**						
Office Hours	4,657	36.1	(34.7-37.5)	3,350	34.9	(33.3-36.6)
Evenings [a]	4,723	36.3	(34.8-37.8)	3,440	36.4	(34.5-38.2)
Weekends [b]	3,626	27.6	(26.5-28.8)	2,738	28.7	(27.3-30.1)
**Was in ED <72hrs prior**	514	3.8	(2.8-5.1)	389	3.8	(2.7-5.3)
**Visit severity**						
**Triage level**						
Urgent or Emergent	5,174	38.9	(35.8-42.2)	3,450	35.5	(32.4-38.7)
Semi-Urgent or Nonurgent	4,418	33.7	(31.0-36.6)	3,519	36.6	(33.8-39.4)
Unknown or Triage not Performed	3,414	27.3	(23.3-31.8)	2,559	28.0	(23.7-32.7)
**Arrived by ambulance**	1,075	8.6	(7.9-9.4)	889	9.3	(8.4-10.2)
**Care delivered**						
**Any Imaging use**	6,178	47.8	(45.9-49.6)	4,648	49.0	(47.2-50.9)

© 2018 Chou S-C et al. *JAMA Network Open.*

CT/MRI	1,942	15.4	(14.2-16.6)	1,478	15.8	(14.6-17.2)
Ultrasound	593	4.4	(3.9-5.1)	380	3.8	(3.2-4.5)
**Total diagnostic services[c]**						
0	3,519	26.0	(24.4-27.6)	2,728	27.6	(26.0-29.2)
1	3,243	25.8	(24.5-27.1)	2,780	29.5	(28.1-31.0)
2-4	2,976	24.0	(22.5-25.5)	2,029	22.5	(21.0-24.0)
≥5	3,268	24.3	(22.3-26.4)	1,991	20.4	(18.5-22.4)
**IV fluids**	3,389	25.7	(23.7-27.8)	2,068	21.7	(19.8-23.8)
**Disposition**						
Admit (observation or inpatient)	637	4.5	(3.9-5.3)	478	4.6	(3.9-5.4)
Critical Care, OR, or Catheterization Lab	114	0.8	(0.6-1.0)	108	0.9	(0.7-1.3)
Discharge	11,578	90.1	(89.0-91.2)	8,470	89.8	(88.5-90.9)
Transfer	98	0.7	(0.5-0.9)	79	0.8	(0.5-1.1)
Left AMA	93	0.6	(0.5-0.8)	75	0.7	(0.5-1.0)
Died upon Arrival or in ED	1			3		
Unknown	599	4.1	(3.3-5.0)	423	4.1	(3.3-5.0)

[a] Evenings defined as 5pm Monday through Friday to 8am the next day.
[b] Weekend defined as 8AM on Saturday to 8AM Monday.
[C] Total diagnostic services include any blood or urine testing, electrocardiogram, or any imaging.
Abbreviations: ED, Emergency department; CI, confidence interval; IV, intravenous; OR, Operating room; AMA, against medical advice **Note**: Weighted proportion not calculated if cells had fewer than 30 records, which create unstable estimates.

© 2018 Chou S-C et al. *JAMA Network Open.*

eTable 4. Frequencies and Proportions of Top 20 Diagnoses among Denial Diagnosis Visits

	Denial Diagnosis Visits (n=4,440)		
Diagnosis	Unweighted Count	Weighted Proportion (95% CI), %	
Sprain and strains of ankle, unspecified site	204	4.2	(3.5-5.1)
Urinary tract infection, site not specified	185	4.1	(3.2-5.2)
Lumbago	181	4.0	(3.2-5.0)
Pain in limb	158	3.7	(3.0-4.5)
Acute pharyngitis	158	3.4	(2.8-4.2)
Unspecified disorder of the teeth and supporting structures	102	3.1	(2.2-4.5)
Acute bronchitis	112	2.3	(1.8-3.0)
Allergy, unspecified	89	2.0	(1.5-2.6)
Essential hypertension, unspecified	83	1.9	(1.4-2.4)
Lumbar sprains and strains	68	1.7	(1.1-2.6)
Cough	63	1.5	(1.0-2.2)
Knee, leg, ankle, and foot injury, other rand unspecified	47	1.5	(0.9-2.4)
Sprain and strains of shoulder and upper arm, unspecified site	61	1.4	(1.0-2.0)
Pain in joint, shoulder	60	1.4	(1.1-2.0)
Pain in joint, lower leg	67	1.3	(1.0-1.8)
Cellulitis of the leg, except foot	64	1.3	(0.9-1.8)
Rash and other nonspecific skin eruption	57	1.2	(0.9-1.7)
Sprains and strains of wrist, unspecified site	56	1.1	(0.8-1.6)
Streptococcal sore throat	49	1.1	(0.8-1.7)

© 2018 Chou S-C et al. *JAMA Network Open.*

eTable 5. Complete list of 329 Reasons for Visit Associated with Denial Diagnosis Visits

≥5 visits within a CCS category among denial diagnosis visits
Abdominal pain, cramps, spasms, NOS
Abnormal color of eyes
Abnormal pulsations and palpitations
Accident, NOS
Allergy, NOS
Animal, human bite
Ankle pain, ache, soreness, discomfort
Anxiety and nervousness
Arm pain, ache, soreness, discomfort
Back pain, ache, soreness, discomfort
Blood in urine (hematuria)
Carbuncle, furuncle, boil, cellulitis, abscess, NEC
Chest pain
Contusions/abrasions/bruises of lower extremity
Contusions/abrasions/bruises of upper extremity
Cough
Depression
Diarrhea
Discoloration or abnormal pigmentation
Earache, pain
Edema
Elbow pain, ache, soreness, discomfort
Excessive sputum
Eye pain
Facial pain
Fever
Flu
Foot and toe pain, ache, soreness, discomfort
Frequency and urgency of urination
General weakness
Groin pain
Hand and finger pain, ache, soreness, discomfort
Head cold, upper respiratory infection (coryza)
Headache, pain in head
Hip pain, ache, soreness, discomfort
Hypertension
Infection of skin of leg, foot, or toe
Injury, Multipleiple or unspecified
Injury, other and unspecified of head, neck, and face
Injury, other and unspecified, of ankle
Injury, other and unspecified, of arm
Injury, other and unspecified, of back
Injury, other and unspecified, of elbow
Injury, other and unspecified, of foot and toe(s)
Injury, other and unspecified, of hand and finger(s)
Injury, other and unspecified, of knee
Injury, other and unspecified, of leg
Injury, other and unspecified, of shoulder
Injury, other and unspecified, of wrist
Insect bite
Insomnia
Knee pain, ache, soreness, discomfort
Laceration/cut of facial area
Laceration/cut of head and neck area
Laceration/cut of lower extremity

© 2018 Chou S-C et al. *JAMA Network Open.*

≥5 visits within a CCS category among denial diagnosis visits
Laceration/cut of upper extremity
Leg pain, ache, soreness, discomfort
Low back pain, ache, soreness, discomfort
Lower abdominal pain, cramps, spasms,
Medication, other and unspecified kinds
Motor vehicle accident, type of injury unspecified
Nasal congestion
Nausea
Neck pain, ache, soreness, discomfort
Nosebleed (epistaxis)
Other and unspecified symptoms referable to eye, NEC
Other diseases of the skin
Other urinary dysfunctions
Pain during pregnancy
Pain, unspecified
Painful urination
Pelvic pain
Plugged feeling in ear
Rib pain
Shortness of breath
Shoulder pain, ache, soreness, discomfort
Side pain, flank pain
Skin itching
Skin lesion
Skin rash
Sprain and strain of ankle
Suture - insertion, removal
Swelling of eyes
Swelling of leg
Symptoms of onset of labor
Symptoms of teeth and gums
Throat pain
Throat soreness
Toothache
Urinary tract infection, NOS
Uterine and vaginal bleeding
Vertigo - dizziness
Vomiting
Wrist pain, ache, soreness, discomfort

≥2 Visits within a CCS category among denial diagnosis visits
Abdominal mass or tumor
Abnormal sensation (paresthesia)
Adverse effect of medication
Back cramps, contractures, spasms
Back symptoms
Blank
Bleeding, Multipleiple or unspecified sites
Burn, all degrees, to extremities
Chest discomfort, pressure, tightness
Chills
Contusion, abrasion, bruise, site unspecified
Contusions/abrasions/bruises of Head, neck, and face
Convulsions
Cramps, spasms, site unspecified
Diagnosed complications of pregnancy and puerperium
Difficulty in swallowing (dysphagia)

© 2018 Chou S-C et al. *JAMA Network Open.*

≥2 Visits within a CCS category among denial diagnosis visits
Diminished hearing
Diminished vision
Discharge from eye
Discharge from eye--Pus, matter, white discharge, matted
Disorders of motor functions
Dressing, bandage - application, change
Excessively heavy (menorrhagia) menstrual flow
Eye burning
Eye itching
Foreign body in digestive tract
Foreign body in eye
Foreign body in other and unspecified sites
Fracture/dislocation of arm
Fracture/dislocation of hand and fingers
General ill feeling
General or unspecified nonviral infection
Gum pain
Hand and finger symptoms
Hoarseness, loss of voice
Infection of skin of arm, hand, or finger
Infection, inflammation, swelling of eyelids
Infections of skin, NOS
Inflammation and swelling of nose
Ingestion, inhalation, or exposure to potentially poisonous products
Injury, other and unspecified, of chest and abdomen
Injury, other and unspecified, of eye
Labored or difficult breathing (dyspnea)
Laceration/cut, site unspecified
Leg symptoms
Loss of feeling (anesthesia)
Lump, mass, tumor of back
Lump, mass, tumor of foot and toe
Medical counseling, NOS
Migraine headache
Mouth pain, burning, soreness
Mouth ulcer, sore
Other symptoms/problems relat to psychological and mental disorders, NEC
Other and unspecified infectious and parasitic diseases
Other and unspecified symptoms referable to digestive system
Other endocrine, nutritional, metabolic and immunity diseases
Other musculoskeletal symptoms
Other specific therapeutic procedures, NEC (cont.)
Other symptoms referable to the cardiovascular/lymphatic system, NEC
Other symptoms referable to skin
Other symptoms referable to urinary tract
Pain or soreness of breast
Physical examination required for employment
Pinkeye
Postoperative visit
Pregnancy, unconfirmed
Problems of pregnancy
Progress visit, NOS
Puncture wound, site unspecified
Retention of urine
Shoulder symptoms
Sinus congestion
Sinus inflammation, infection

© 2018 Chou S-C et al. *JAMA Network Open.*

≥2 Visits within a CCS category among denial diagnosis visits
Sinus pain and pressure
Spotting, bleeding during pregnancy
Sprain and strain of wrist
Sprain or strain, other and unspecified
Stomach and abdominal pain, cramps and spasms
Complications of surgical or medical procedures and treatments
Swelling of ankle
Swelling of arm
Swelling of elbow
Swelling of foot and toe
Swelling of hand and finger
Swelling of knee
Swelling of skin
Symptoms of fluid abnormalities
Throat swelling
Tiredness, exhaustion
Tongue inflammation, infection, swelling
Unspecified joint pain, ache, soreness, discomfort
Unusual color or odor of urine
Upper abdominal pain, cramps, spasms
Upper respiratory infections except tonsillitis
Vaginal discharge
Vaginal pain
Violence, NOS
Wrist symptoms

<2 visits within a CCS category among denial diagnosis visits
Abdominal swelling, NOS
Abnormal involuntary movements
Abnormal sensations of the eye
Adverse effects, other and unspecified
Anal-rectal bleeding
Anemia
Arm symptoms
Arthritis
Asthma
Bladder infection
Blindness and half vision
Blood in stool (melena)
Breathing problems, NEC
Burn, all degrees, to head, neck, and face
Burn, all degrees, to trunk area
Congestion in chest
Constipation
Coughing up blood
Dental abscess
Diabetes mellitus
Diet and nutritional counseling
Discharge from ear
Discharge from eye--bleeding
Discharge from eye--Tearing, watering (lacrimation)
Diseases of the intestine and peritoneum
Diseases of the thyroid gland
Ear infection
Earache, or ear infection
Economic problem
Elbow symptoms

© 2018 Chou S-C et al. *JAMA Network Open.*

<2 visits within a CCS category among denial diagnosis visits
Entry of 'none' or 'no complaint'
Excessive sweating, perspiration
Fainting (syncope)
Fibrocystic and other diseases of breast
Foot and toe symptoms
For other findings of blood tests
For results of blood glucose tests
For results of EKG
Foreign body in respiratory tract
Fracture, other and unspecified
Fracture/dislocation of ankle
Fracture/dislocation of head and face
Fracture/dislocation of wrist
Functional psychoses
Fungus infections (mycoses)
General medical examination
General psychiatric or psychological examination (mental health)
Growths or mass of ears
Hand and finger cramps, contractures, spasms
Hay fever
Hearing dysfunctions
Hip symptoms
Hysterical behavior
Illegible entry
Inadequate data base
Increased heartbeat
Inflammatory diseases of the eye
Injections
Injury, other and unspecified, of hip
Intermenstrual bleeding (metrorrhagia)
Internal prosthetic device
Irregular heartbeat
Itching of scalp
Kidney infection
Kidney pain
Laceration/cut of trunk area
Late effects of an old injury
Leg cramps, contractures, spasms
Limitation of movement, stiffness of knee
Limitation of movement, stiffness of neck
Limitation of movement, stiffness of arm
Lump or mass of breast
Lump, mass, tumor of arm
Lump, mass, tumor of wrist
Navel problems
Nonarticular rheumatism
Organic psychoses
Other and unspecified symptoms referable to the ears, NEC
Other disease of circulatory system
Other diseases of female reproductive system
Other growths of skin
Other special examination
Other symptoms of male reproductive system
Other symptoms of nose
Other symptoms of the heart
Other symptoms referable to the female reproductive system
Other symptoms referable to the nervous system

© 2018 Chou S-C et al. *JAMA Network Open.*

<2 visits within a CCS category among denial diagnosis visits
Other symptoms referable to the respiratory system
Other vaginal symptoms
Pain, aching, soreness, tenderness, painful erection of penis
Pain, aching, tenderness of the scrotum and testes
Pain, specified site not referable to a specific body system
Painful menstruation (dysmenorrhea)
Pelvic pressure or dropping sensation
Pelvic symptoms
Penile discharge
Physical examination for extracurricular activities
Police involvement in outpatient visit circumstances
Prenatal examination, routine
Preoperative visit for specified/unspecified types of surgery
Problems, complaints, NEC
Prophylactic inoculations
Psoriasis
Puncture wound of lower extremity
Puncture wound of upper extremity
Rape
Skin irritations, NEC
Skin pain
Sprain and strain of back
Sprain and strain of knee
Streptococcal infection
Sunburn, windburn
Swelling of neck
Swelling, inflammation of the scrotum and testes
Swollen or enlarged glands
Symptoms of penis
Symptoms of unspecified joints
Symptoms of unspecified muscles
Symptoms referable to lips
Symptoms referable to mouth
Symptoms referable to nails
Symptoms referable to throat
Symptoms referable to tongue
Tongue pain
Tonsillitis
Unspecified muscle cramps, contractures, spasms
Unspecified muscle pain, ache, soreness, discomfort
Urinary tract instrumentation and catheterization
Vaginal itching, burning
Vasomotor symptoms-hot flashes
Viral diseases
Vision dysfunctions
Vulvar disorders
Weakness (neurologic)
Weakness of knee
Wheezing
Worker's Compensation examination

Abbreviations: NEC, none elsewhere classified; NOS, none otherwise specified.

© 2018 Chou S-C et al. *JAMA Network Open.*

eTable 6. Denial Symptom Visits as Defined by Primary Symptoms Present in ≥2 or ≥5 Denial Diagnosis Visits

	2011-2015 National Hospital Ambulatory Medical Care Survey - ED Subsample					
	Denial Symptom Visits ≥2 visits (n=24,882)			Denial Symptom Visits ≥5 Visits (n=21,013)		
	Unweighted Count	Weighted % (95% CI)		Unweighted Count	Weighted % (95% CI)	
**Demographics**						
**age (category)**						
15-24 years old	5,490	22.1	(21.3-22.9)	4,620	22.1	(21.2-23.1)
25-44 years old	10,053	40.1	(39.2-40.9)	8,479	40.0	(39.1-41.0)
45-64 years old	9,339	37.8	(37.0-38.7)	7,914	37.8	(36.9-38.8)
**Female**	14,362	57.9	(56.9-58.9)	12,037	57.7	(56.7-58.7)
**Race**						
White, Non-Hispanic	17,483	68.7	(66.2-71.1)	14,706	68.3	(65.9-70.7)
Black, Non-Hispanic	3,837	17.6	(15.4-20.1)	3,272	17.8	(15.5-20.3)
Hispanic	2,647	10.8	(9.4-12.3)	2,256	11	(9.6-12.5)
Other, Non-Hispanic	915	3.0	(2.4-3.6)	779	2.9	(2.4-3.5)
**Region**						
Northeast	5,173	18.8	(15.0-23.4)	4,344	18.7	(15.0-23.1)
Midwest	6,320	25.1	(20.1-30.8)	5,342	25.3	(20.2-31.1)
South	8,164	36.1	(30.9-41.6)	6,944	36	(30.8-41.5)
West	5,225	20.0	(16.1-24.6)	4,383	20	(16.1-24.7)
**Visit timing**						
**Follow-up Visit**	1,098	4.2	(3.7-4.7)	893	4.0	(3.6-4.6)
**Time of day**						
Office Hours	8,700	34.7	(33.8-35.6)	7,415	35.0	(34.1-36.0)
Evenings[a]	9,236	37.4	(36.5-38.4)	7,746	37.3	(36.3-38.3)
Weekends[b]	6,946	27.9	(27.1-28.7)	5,852	27.7	(26.9-28.5)
**Was in ED <72hrs prior**	996	3.8	(2.9-5.0)	830	3.8	(2.8-5.0)
**Visit severity**						
**Triage level**						
Immediate	10,870	43.2	(40.2-46.4)	9,045	42.5	(39.4-45.7)
Emergent	7,360	28.9	(26.8-31.2)	6,413	29.8	(27.6-32.2)
Unknown or Triage not Performed	6,652	27.8	(24.1-31.9)	5,555	27.7	(23.8-31.9)
**Arrived by ambulance**	2,480	10.3	(9.8-10.9)	1,999	9.9	(9.3-10.5)
**Care delivered**						

© 2018 Chou S-C et al. *JAMA Network Open.*

**Any Imaging use**	12,778	52.0	(50.4-53.6)	11,187	53.6	(52.0-55.2)
CT/MRI	4,790	19.6	(18.4-20.8)	4,145	20	(18.8-21.3)
Ultrasound	1,268	5.0	(4.5-5.5)	1,012	4.7	(4.2-5.2)
**Total diagnostic services[c]**						
0	6,437	24.9	(23.6-26.2)	5,276	24.4	(23.1-25.7)
1	5,669	23.2	(22.1-24.2)	5,022	24.2	(23.1-25.3)
2-4	5,517	23.5	(22.3-24.8)	4,628	23.4	(22.0-24.7)
≥5	7,259	28.4	(26.5-30.4)	6,087	28.1	(26.2-30.1)
**IV fluids**	7,537	30.1	(28.0-32.2)	6,351	29.9	(27.9-32.0)
**Disposition**						
Admit (observation or inpatient)	2,189	8.2	(7.3-9.2)	1,804	7.9	(7.1-8.9)
Critical Care, OR, or Catheterization Lab	564	2.0	(1.7-2.4)	466	2.1	(1.7-2.4)
Discharge	20,874	85.2	(83.9-86.4)	17,727	85.8	(84.5-87.0)
Transfer	408	1.4	(1.2-1.7)	302	1.3	(1.0-1.5)
Left AMA	372	1.5	(1.3-1.7)	303	1.4	(1.2-1.7)
Died upon Arrival or in ED	5			3		
Unknown	1,034	3.7	(3.1-4.4)	874	3.6	(3.0-4.3)

[a] Evenings defined as 5pm Monday through Friday to 8am the next day.
[b] Weekend defined as 8AM on Saturday to 8AM Monday.
[C] Total diagnostic services include any blood or urine testing, electrocardiogram, or any imaging.
Abbreviations: ED, Emergency department; CI, confidence interval; IV, intravenous; OR, Operating room; AMA, against medical advice **Note**: Weighted proportion not calculated if cells had fewer than 30 records, which create unstable estimates.

© 2018 Chou S-C et al. *JAMA Network Open.*

eTable 7. Denial Symptom Visits, By Data Years

	National Hospital Ambulatory Medical Care Survey - ED Subsample					
**Data Year**	**2011-2015 (n=24,882)**			**2014-2015 (n=7,878)**		
	Unweighted Count	Weighted % (95% CI)		Unweighted Count	Weighted % (95% CI)	
**Demographics**						
**age (category)**						
15-24 years old	5,490	22.1	(21.3-22.9)	1,718	21.8	(20.4-23.3)
25-44 years old	10,053	40.1	(39.2-40.9)	3,249	41.3	(39.7-42.9)
45-64 years old	9,339	37.8	(37.0-38.7)	2,911	36.9	(35.3-38.4)
**Female**	14,362	57.9	(56.9-58.9)	4,720	60.2	(58.5-61.9)
**Race**						
White, Non-Hispanic	17,483	68.7	(66.2-71.1)	5,461	66.8	(62.3-71.0)
Black, Non-Hispanic	3,837	17.6	(15.4-20.1)	1,323	19.9	(16.0-24.4)
Hispanic	2,647	10.8	(9.4-12.3)	848	10.6	(8.6-13.0)
Other, Non-Hispanic	915	3.0	(2.4-3.6)	246	2.7	(2.0-3.6)
**Region**						
Northeast	5,173	18.8	(15.0-23.4)	1,562	18.1	(13.5-23.9)
Midwest	6,320	25.1	(20.1-30.8)	2,055	29.5	(22.3-37.9)
South	8,164	36.1	(30.9-41.6)	2,708	35.9	(29.8-42.4)
West	5,225	20.0	(16.1-24.6)	1,553	16.5	(12.6-21.4)
**Visit timing**						
**Follow-up Visit**	1,098	4.2	(3.7-4.7)	357	4.4	(3.7-5.3)
**Time of day**						
Office Hours	8,700	34.7	(33.8-35.6)	2,760	35.7	(34.3-37.1)
Evenings[a]	9,236	37.4	(36.5-38.4)	2,977	36.5	(34.7-38.3)
Weekends[b]	6,946	27.9	(27.1-28.7)	2,141	27.8	(26.5-29.1)
**Was in ED <72hrs prior**	996	3.8	(2.9-5.0)	356	5.0	(3.0-8.2)
**Visit severity**						
**Triage level**						
Immediate	10,870	43.2	(40.2-46.4)	3,187	39.2	(33.8-44.9)
Emergent	7,360	28.9	(26.8-31.2)	2,379	28.6	(24.5-33.0)
Unknown or Triage not Performed	6,652	27.8	(24.1-31.9)	2,312	32.2	(25.1-40.3)
**Arrived by ambulance**	2,480	10.3	(9.8-10.9)	714	9.5	(8.7-10.5)
**Care delivered**						
**Any Imaging use**	12,778	52.0	(50.4-53.6)	4,066	52.3	(49.6-55.0)
CT/MRI	4,790	19.6	(18.4-20.8)	1,519	19.4	(17.4-21.6)
Ultrasound	1,268	5.0	(4.5-5.5)	425	5.7	(4.9-6.6)

© 2018 Chou S-C et al. *JAMA Network Open.*

**Total diagnostic services**[c]						
0	6,437	24.9	(23.6-26.2)	1,989	24.2	(22.1-26.4)
1	5,669	23.2	(22.1-24.2)	1,778	23.0	(21.1-25.0)
2-4	5,517	23.5	(22.3-24.8)	1,862	24.6	(22.4-26.9)
≥5	7,259	28.4	(26.5-30.4)	2,249	28.2	(25.1-31.6)
**IV fluids**	7,537	30.1	(28.0-32.2)	2,289	28.1	(24.4-32.1)
**Disposition**						
Admit (observation or inpatient)	2,189	8.2	(7.3-9.2)	585	6.9	(5.8-8.3)
Critical Care, OR, or Catheterization Lab	564	2.0	(1.7-2.4)	143	1.7	(1.3-2.2)
Discharge	20,874	85.2	(83.9-86.4)	6,898	89.3	(87.7-90.7)
Transfer	408	1.4	(1.2-1.7)	110	0.9	(0.7-1.3)
Left AMA	372	1.5	(1.3-1.7)	105	1.2	(0.9-1.5)
Died upon Arrival or in ED	5			0		
Unknown	1,034	3.7	(3.1-4.4)	180	1.7	(1.2-2.3)

[a] Evenings defined as 5pm Monday through Friday to 8am the next day.
[b] Weekend defined as 8AM on Saturday to 8AM Monday.
[C] Total diagnostic services include any blood or urine testing, electrocardiogram, or any imaging.
Abbreviations: ED, Emergency department; CI, confidence interval; IV, intravenous; OR, Operating room; AMA, against medical advice **Note**: Weighted proportion not calculated if cells had fewer than 30 records, which create unstable estimates.

© 2018 Chou S-C et al. *JAMA Network Open.*
